# Reinforcing implementation intentions with imagery increases physical activity habit strength and behaviour

**DOI:** 10.1111/bjhp.12795

**Published:** 2025-03-18

**Authors:** Alison Divine, Sarah Astill

**Affiliations:** ^1^ School of Biomedical Sciences, Faculty of Biological Sciences University of Leeds Leeds UK

**Keywords:** habit strength, imagery, implementation intentions, physical activity

## Abstract

**Objectives:**

Habits may enhance the maintenance of physical activity. The aim of this study is to examine if reinforcing implementation intentions increases habit strength and thus physical activity.

**Design:**

Participants (*N* = 186) were randomized into one of three intervention conditions (imagery vs. implementation intentions vs. combined; implementation intentions and imagery) and a control condition.

**Methods:**

Participants were instructed to engage in a physical activity that they would like to start doing, or do more of, over a four‐week period. Participants completed measures of physical activity, habit strength, and imagery use (imagery and combined conditions only) pre‐intervention, weekly during the intervention, post‐intervention, and a 12‐week follow‐up.

**Results:**

Habit strength increased at week three (*p* < .001) for those in the combined condition, which was maintained through post‐intervention and increased at follow‐up (*Ps > .05*). In the imagery condition, habit strength increased at post‐intervention (*p* = .003) and was maintained at follow‐up. Physical activity increased for the combined condition from week two (*p* < .001) of the intervention, continuing to increase at weeks three (*p* = .003) and four (*p* < .001).

**Conclusions:**

Imagery may be an effective intervention to support habit formation. Reinforcing implementation intentions with mental imagery may support habit formation for physical activity behaviour.

**Statement of Contribution**

**What Is Already Known on this Subject?**

- Action planning and implementation intentions are associated with physical activity habits.
- Mental imagery can increase physical activity behaviour
- Reinforcing implementation intentions with imagery may be beneficial for forming physical activity habits.

**What Does this Study Add?**

- Reinforcing implementation intentions with imagery increased physical activity habit strength 3 weeks into the intervention, which was maintained through to follow‐up.
- Implementation intentions and imagery lead to increases in physical activity behaviour.
- Study findings indicate that reinforcing specific plans via implementation intentions with mental imagery may decrease the time it takes to strengthen physical activity habits, making physical activity more automatic and supporting maintenance of behaviour.

## INTRODUCTION

Moderate to vigorous intensity physical activity performed for 150 min or more per week reduces the risk of at least 25 chronic medical conditions by 20%–30%, including obesity, diabetes, some cancers, and cardiovascular disease (Rhodes, 2017; Watts et al., 2023). However, many adults (36% in the UK) do not meet these recommended levels of physical activity (NHS Digital, 2023). Becoming more active is one of the most frequently adopted health goals; however, most people who intend to be active often face difficulties in changing their behaviour (Rhodes & De Bruijn, 2013). Indeed, 68% of those who intend to start being active and 29% of those who intend to maintain physical activity levels fail to do so (Rhodes & Dickau, 2012). One mechanism for enhancing sustained physical activity is through the formation of habits (Rebar et al., 2016).

Habit is the process through which behaviour is influenced by well‐learned cue–behaviour associations. Over time, as the behaviour is repeated in the same context, people learn to associate certain cues (e.g., location, existing routine events, or time of day) with the initiation of the behaviour (Gardner, 2014). Subsequently, the context becomes sufficient to activate the association between the behaviour and the context, in turn triggering an automatic impulse to perform the habitual behaviour (Gardner, 2014). Gardner and Lally (2018) outline four stages of habit formation. First, a decision is made whether to perform a new behaviour; second, the intention is translated into behaviour by using self‐regulation strategies and volitional control (e.g., implementation intentions). Third, behaviour needs to be repeated, which continues to require self‐regulation and motivation. Fourth, the behaviour is now repeated in a consistent context, resulting in the formation of cue–behaviour links and the formation of habit associations. Moreover, Phillips and Gardner (2016) also distinguish between habitual instigation, habitually deciding to perform a behaviour, and habitual execution, habitually performing the behaviour. Behaviours can be instigated habitually, performed habitually, or a combination of both processes. Recent research indicates those with stronger physical activity habits engage in more physical activity (*r* = .32–.43; Gardner et al., 2011; Rebar et al., 2016; Rhodes, Cox, & Sayar, 2021) with some research indicating that instigation habit is a stronger predictor of physical activity than execution habit (Kaushal et al., 2017, 2018; Phillips & Gardner, 2016).

Despite this, few intervention studies target the formation of physical activity habits. A review of ten physical activity habit formation studies indicated that while there was significant heterogeneity, overall interventions have a significant impact on physical activity habits (Ma et al., 2023). One intervention approach is implementation intentions, which are ‘if‐then’ plans that specify when, where, and how one will obtain their goal (Gollwitzer, 1999) and instigate similar automatic responses as habits (Webb & Sheeran, 2004; Wieber et al., 2015). Specifically, the formation of the ‘if‐then’ plan in implementation intentions creates an association between the situation and the planned response that echoes the situation–response associations that are essential for habitual behaviour (Webb & Sheeran, 2004). Evidence suggests that general action planning and implementation intentions are associated with physical activity habit strength (Fleig et al., 2013; Maltagliati et al., 2022; Schwarzer et al., 2018; van Bree et al., 2016) and may work to increase physical activity behaviour through effects on habit (Rhodes, Cox, & Sayar, 2021). Despite this evidence, research has yet to assess the effectiveness of implementation intentions on the formation of physical activity habits.

Implementation intentions are effective for increasing physical activity with small effect sizes between *d* = .14 (Sheeran et al., 2024) and *d* = .31 (Bélanger‐Gravel et al., 2013; Fennis et al., 2011; Gollwitzer & Sheeran, 2006) which are sustained after no‐contact follow‐up periods (Bélanger‐Gravel et al., 2013). However, there is substantial heterogeneity in effect size (Hagger & Luszczynska, 2014) and when the intention to perform a behaviour is low, implementation intentions have a weak effect on behaviour (Prestwich et al., 2003; Sheeran et al., 2005). The accessibility of performing the behaviour in an implementation intention decreases over time (Tobias, 2009). Prestwich and Kellar (2014) suggest that reinforcing implementation intentions may be especially useful for complex behaviours that require multiple steps to execute, such as engaging in physical activity. Reinforcing implementation intentions involves the application of subsequent strategies during the intervention aimed at enhancing the effectiveness of the implementation intentions. Examples of reinforcement strategies include using reminders of their plans or that it is time to enact the behaviour delivered through text messages (Prestwich et al., 2009, 2010) or face‐to‐face conversations (De Vet et al., 2009; Rodrigues et al., 2013). Other types of reinforcement can include motivational strategies, such as a decision balance sheet that aims to understand gains and losses of enacting their plan (Prestwich et al., 2003). With respect to physical activity behaviour, a meta‐analysis found that studies that used reinforcement of implementation intentions increased physical activity (*d* = .25) compared to groups that did not use reinforcement (*d* = .15; Silva et al., 2018). Most studies reinforced implementation intentions for physical activity with text messages (Prestwich et al., 2009, 2010), face‐to‐face conversations (De Vet et al., 2009; Rodrigues et al., 2013), or telephone reminders (Latimer et al., 2006; Luszczynska et al., 2007; Silva et al., 2018). While reinforcing implementation intentions with reminders shows promise, there are some potential limitations with respect to habit formation. Specifically, reinforcement through reminders can lead to a dependency on that reminder (Renfree et al., 2016) hindering the development of automaticity. For habit formation, strengthening the link between ‘if’ and ‘then’ is essential (Adriaanse et al., 2011). Given that many efforts to change behaviour are characterized by short‐term success followed by relapse (Polivy & Peter Herman, 2002) reinforcing the situation response cues may result in stronger mental associations. As a result, automaticity would occur sooner, reducing the rate of physical activity dropout as behaviour becomes habitual. However, reinforcing implementation intentions has yet to be investigated for habit formation. One relatively simple approach that has been effective in reinforcing implementation intentions to increase behaviour is mental imagery (Knäuper et al., 2009; Knäuper et al., 2011).

Mental imagery involves the representation of an experience in one's mind using multiple senses, without the presence of that actual stimulus (Moran, 2004). Physical activity‐related imagery involves individuals mentally rehearsing the perceptual, motor, and emotional experiences of physical activity. This includes imaging oneself looking forward to being active, getting ready to be active, being physically active, enjoying being active, and achieving certain outcomes (Hall, 1995). A recent meta‐analysis on imagery and health behaviours found that imagery had medium effect sizes for behavioural outcomes, including physical activity (Conroy & Hagger, 2018). Only a few studies have reinforced implementation intention intentions with imagery for goal achievement. These studies have indicated that for changes in diet behaviours, the combination of implementation intentions and imagery is an effective approach (Knäuper et al., 2011).

Reinforcing implementation intentions with imagery may be particularly relevant for habit formation. In line with habit theory, the vivid and realistic representation of the situation and response is thought to strengthen the mental links between actions and outcomes (Escalas & Luce, 2004), which, in turn, may help develop habits. To date, there has been no experimental investigation into the effects of implementation intentions reinforced through mental imagery for the formation of physical activity habits. While there have been a few studies that have reinforced implementation intentions with imagery for goal‐directed behaviours (e.g., Knäuper et al., 2009, 2011) this study explicitly tests whether instructing participants to imagine key elements of their implementation intentions increases physical activity habit strength. The primary aim of this preliminary study was to examine the effects of combining implementation intentions and imagery on physical activity habit strength and on subsequent self‐reported physical activity levels.

While implementation intentions should result in increases in automaticity (Gollwitzer, 1999), a recent review indicated that implementation intentions had a small effect on physical activity behaviour (*d* = .14; Sheeran et al., 2024). A key factor determining the effectiveness of implementation intentions is the cognitive accessibility of the context–behaviour situation (Lally & Gardner, 2013; Tobias, 2009). Active reinforcement through mental imagery can increase the accessibility of the cue and behaviour responses (Tobias, 2009), which means that an individual should be more likely to act when the cue is encountered, increasing automaticity and frequency of behaviour. Indeed, Gollwitzer and Sheeran (2006) proposed that mental imagery may increase the effectiveness of implementation intentions. In addition, motivation had a large moderating effect (*d* = .79) of implementation intentions on behaviour (Sheeran et al., 2024). Imagining positive affective experiences (e.g., looking forward to being active, feeling good during activity, and successfully completing activity) may be rewarding and promote intrinsic motivation and positive affect. Mental imagery has been referred to as a ‘motivation amplifier’ and can enhance motivation, anticipated affect, and reward (Renner et al., 2019). According to habit theory, enhancing intrinsic motivation, positive affect, and rewards can strengthen the impact of repetition on habit formation by reinforcing the cue–response relationship (Gardner & Lally, 2018). Thus, reinforcing implementation intentions with mental imagery may enhance automaticity and habit formation.

Based on the above findings, we hypothesized that participants in the implementation intention and imagery (combined) condition would have stronger physical activity habit strength and physical activity levels post‐intervention and at follow‐up than those in other conditions with the lowest habit strength and physical activity in the control condition.

## METHODS

### Participants

Participants were recruited from Prolific Academic and paid £4.50 for their time, and the study was run on Gorilla Experiment Software. Inclusion criteria were that participants were 18 years or older. Participants provided informed consent, and ethical approval was obtained from the Faculty of Biological Sciences Research Ethics Board.

An a priori sample size calculation was completed using G*Power. Effect size was chosen based on meta‐analyses of the impact of imagery (Conroy & Hagger, 2018) implementation intentions on physical activity (Bélanger‐Gravel et al., 2013) and interventions that impact physical activity habit strength (Feil et al., 2021) which ranged from .24–.31. The lower effect size was chosen as a conservative measure and to ensure sufficient power. With an effect size of 0.24, power of 0.80, the minimum sample size needed was 84 participants (21 in each group). To account for attrition, which averages 20% in internet‐based physical activity interventions (Davies et al., 2012) we aimed to recruit a minimum of 100 participants (25 per group).

### Procedure

**FIGURE 1:**
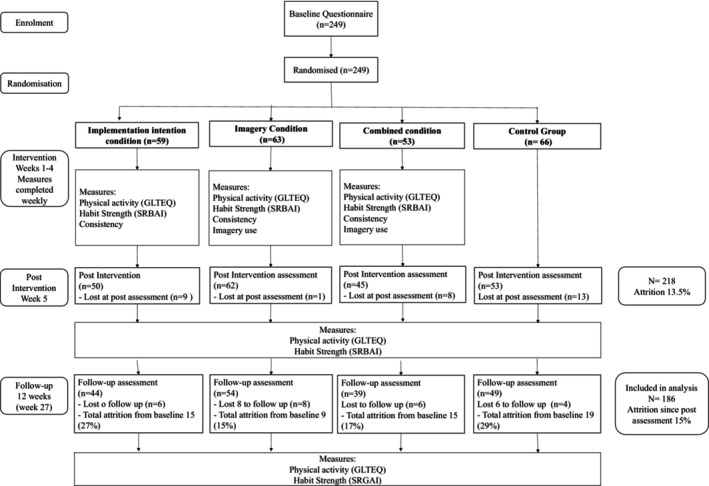
Flow diagram of the study.

After completing demographic and baseline assessments, participants were randomized using the randomizer node on Gorilla Software into one of four conditions: (1) imagery, (2) implementation intentions, (3) imagery and implementation intentions (combined) (4) control. Participants in the three intervention conditions were automatically sent an email every 7 days via the online platform with the link to log into the study. They were then prompted to complete their respective intervention and weekly measures of physical activity, habit strength, behavioural consistency, and those in the imagery and combined conditions also reported their imagery use. The intervention lasted for 4 weeks. Participants in the no‐contact control condition were not given any instructions with respect to physical activity and completed baseline, post, and follow‐up assessments only. All participants were invited to complete post‐assessments at 5 weeks and a follow‐up assessment 12 weeks later (week 27). A flow diagram of the study is presented in Figure 1.

### Conditions

Those randomized into the *imagery condition* completed a measure of imagery ability, were provided information on the effectiveness of imagery for physical activity, and received instructions on how to complete the imagery sessions. An imagery script was provided within the software that guided their imagery and asked participants to create images of the experience of being active in their minds, including preparing to be active, engaging in the activity, and experiencing the movements, sounds, sights, and emotions as they would if they were physically completing the activity. Participants logged into the study to complete an imagery session once per week and were asked to complete two additional imagery sessions on their own. They received a copy of the imagery script via email. Each imagery session took approximately 3 minutes to complete.

In the *implementation intention condition*, participants were asked to create an implementation intention that contained three components: (1) a physical activity they do not already do or want to do more of, (2) could be done in response to a daily event (cue) and (3) a cue that occurs once a day every day (e.g., dinner). Participants were asked to log into the study every week and review their implementation intention.

In the *implementation intention and imagery condition (combined condition)*, participants created an implementation intention as described above. Participants logged into the study once per week to review their implementation intention and engage in an imagery session. Participants followed the same procedures as those in the imagery only condition; however, the script for the combined condition was targeted to their implementation intentions. This was done automatically within the software that carried forward their implementation intention and inserted it into the imagery script. Imagery instructions and imagery scripts for the imagery only condition and combined condition are available in File S1.

### Measures

#### Demographics

Participants self‐reported their age, gender, ethnicity, height, weight, and level of education.

#### Physical activity

Physical activity was assessed using the Godin Leisure‐Time Questionnaire (GLTEQ; Godin, 2011). Participants reported their typical frequency of weekly leisure‐time physical activity in bouts of 15 min or more for vigorous (Heart beating rapidly, sweating. e.g. Running), moderate (Not exhausting, light perspiration. e.g. Fast walking) intensities. Weekly frequencies of vigorous and moderate activities were multiplied by nine and five respectively, with the products being summed to produce an overall Leisure Index Score (LIS).

#### Habit strength

Habit Strength was assessed using the Self Report Behavioural Automaticity Index (SRBAI; Gardner et al., 2012). The SRBAI consists of 4 items on a 5‐point Likert scale ranging from 1, “*strongly disagree*” to 5, “*strongly agree*.” Following the stem: “*Physical activity is something…*” and participants rate their agreement with the answers: “*I do automatically*”, “*I do without having to consciously remember*”, “*I do without thinking*” and “*I start doing it before I realise I am doing it*”. The SRBAI displays high reliability with a Cronbach's between *α* = .96–.98 across all time points.

#### Imagery ability

To ensure that any observed effects for the imagery were not due to differential ease of imagery, participants in the imagery and combined conditions completed a measures of imagery ability using the Vividness of Movement Imagery Questionnaire 2 (VMIQ‐2; Roberts et al., 2008) which assess ease of imaging movements. An example item asks participants to indicate how vivid the image of walking is on a 5‐point Likert scale from 1 *“no image at all, you only “know” you are thinking of a skill”* to 5 *“perfectly clear and as vivid as normal vision or feel of movement*.” The VMIQ‐2 has demonstrated factorial validity, and test–retest reliability (Roberts et al., 2008).

### Intervention manipulation and fidelity checks

#### Adherence

To assess adherence and engagement in the intervention, the number of weeks participants logged into the study to complete the weekly questionnaires was recorded, and for those in the imagery and combined conditions, participants were asked each week to indicate which days of the week they completed the imagery sessions.

#### Consistency of behaviour

In line with Kaushal et al. (2017) consistency was assessed with 1 item “How consistently were you active at the same time each day?”. The options ranged on a 4‐point Likert scale with 0 = not consistent, always at random times to 3 = always consistent.

#### Implementation intention fidelity

Prior to the intervention starting, a researcher (AD) checked all implementation intentions written by those in the implementation intention and combined conditions to ensure that they followed the instructions. Those that did not were asked to rewrite their implementation intentions in accordance with the ‘if‐then’ format. This check was completed once at the beginning of the intervention. Participants were not asked to revise or rewrite their plans at any point after their initial implementation intention was written.

## STATISTICAL ANALYSIS

Those who completed all time points were included in the study. Missing data points were either carried forward (e.g., physical activity data) or, when one item was missing on the SRBAI, a substitution score was used; this was the individual mean score of the completed items of the SRBAI. Differences in demographic variables between conditions were assessed with one‐way Analysis of Variance (ANOVA) or Chi‐square where appropriate. Means (*SD*) and frequencies are calculated for adherence to imagery sessions.

### Intervention manipulations and fidelity checks

The number of imagery sessions was assessed using frequencies and percentages. Differences in imagery ability (internal, external and kinaesthetic imagery ability) were assessed using a one‐way ANOVA between the imagery and combined conditions. A mixed Repeated Measures Analysis of Variance (RM ANOVA) comprising consistency measures at each of the intervention weeks (week 1, 2, 3, and 4) as a within‐subject factor (time) and condition (imagery, implementation, or combined) as between‐subject factor was used to assess the differences in consistency of behaviour.

### Effects of condition on habit strength and physical activity

Two mixed RM ANOVAs were used to assess changes in habit strength and physical activity LIS scores from baseline, post‐intervention, and follow‐up across all conditions. A further two separate mixed RM ANOVAs were run to assess weekly, post‐intervention, and follow‐up changes in habit strength and physical activity LIS scores across the intervention conditions only. In each model, there was one between‐subject factor (condition) and one within‐subject factor (time). Significant main effects are investigated using pairwise comparisons with Bonferroni adjustments to control for multiple comparisons, and significant interactions were further examined using paired‐samples t‐tests.

## RESULTS

### Participant characteristics

A total of 249 (*M* = 86 (35%), *F* = 160 (64%), 3 nonbinary (1%)) participants completed baseline measures (Mean age = 45.12 ± 14.19 years) and were randomized into one of the four conditions. Of the 249 participants, 186 completed post‐intervention assessment 5 weeks after baseline and follow‐up measures 12 weeks later (week 27; attrition of 28%). Most participants included in the analysis identified as female (64%), had a bachelor's degree (39%), were Caucasian (84%) and had an average body mass index (BMI) of (29.05 ± 7.12). Those who dropped out were significantly younger (Dropouts: age 40.71 ± 14.05 years of age, completed 46.61 ± 13.96 years of age *t* (247) = 2.89, *p* = .002) and had higher habit strength for physical activity (drop out: 2.22 ± 1.09, completed: 1.90 ± .95, *t* (247) = −2.20, *p* = .014). No differences for the demographic variables were found between the conditions for age (*F*_(3,182)_ = .68, *p* = .563), BMI (*F*_(3,182)_ = 1.27, *p* = .286), gender (*X* ^2^ = 1.45, *p* = .963), education (*X* ^2^ = 7.45, *p* = .826), or ethnicity (*X* ^2^ = 35.17, *p* = .645). There were no differences in baseline levels of physical activity (*F*_(3,245)_ = .19, *p* = .905) or habit strength (*F*_(3,245)_ = .69, *p* = .447) between conditions. Complete participant characteristics are in Table 1.

**TABLE 1:** Demographic data, intervention adherence, physical activity and habit strength scores for the whole sample and by intervention condition.

Variable	Total sample (*n* = 186)	Control (*n* = 49)	Imagery (*n* = 54)	Implementation intention (*n* = 44)	Combined (*n* = 39)
Age (Mean ± *SD*)	46.61 ± 13.96	48.18 ± 12.61	44.57 ± 14.53	46.32 ± 15.06	47.77 ± 13.64
Gender *n* (%) female	120 (65%)	31 (63%)	32 (59%)	31 (71%)	26 (67%)
Education *n* (%)*					
Secondary School	56 (32%)	15 (33%)	18 (38%)	10 (24%)	13 (34%)
College	12 (7%)	3 (7%)	4 (9%)	3 (7%)	2 (5%)
Technical	21 (12%)	8 (17%)	3 (6%)	7 (17%)	3 (8%)
Bachelors	66 (38%)	14 (30%)	17 (36%)	18 (10%)	17 (45%)
Masters	18 (10%)	6 (13%)	5 (11%)	4 (10%)	3 (8%)
Ethnicity: Caucasian *n* (%)	162 (87%)	47 (96%)	45 (83%)	36 (82%)	34 (87%)
BMI Mean ± *SD* *	29.05 ± 7.12	30.42 ± 8.29	27.71 ± 5.78	28.80 ± 6.16	29.53 ± 8.33
Physical activity (LIS)					
Baseline	17.87 ± 6.64	17.10 ± 6.34	18.55 ± 7.78	18.41 ± 6.88	17.14 ± 4.67
Week 1			1.84 ± 1.10	2.18 ± .94	2.07 ± 1.00
Week 2			1.94 ± 1.25	2.14 ± .98	2.21 ± 1.03
Week 3			1.86 ± 1.22	2.18 ± 1.09	3.13 ± .70
Week 4			1.83 ± 1.37	2.21 ± 1.09	3.18 ± .10
Post	11.16 ± 14.39	8.19 ± 11.85	10.53 ± 15.44	12.84 ± 13.09	13.51 ± 16.80
Follow‐up	12.70 ± 16.01	12.95 ± 16.01	11.21 ± 18.09	12.95 ± 13.09	18.6 ± 16.14
Habit Strength					
Baseline	1.90 ± .93	1.70 ± .72	1.85 ± .92	2.04 ± 1.01	2.03 ± .04
Post	2.30 ± 1.10	1.79 ± .71	2.20 ± 1.27	2.19 ± 1.10	3.22 ± .67
Follow‐up	2.39 ± 1.05	1.82 ± .69	2.38 ± 1.11	2.14 ± 1.03	3.36 ± .63
Imagery ability					
Kinaesthetic			3.11 ± .66		3.26 ± .68
Visual external			3.70 ± .64		3.58 ± .99
Visual internal			3.85 ± .79		3.77 ± .97
Consistency			.90 ± .78	1.18 ± .69	1.22 ± .81

*Note*: Demographic variables with missing data are indicated with an*. Education had a total of 13 missing data points: three in the control condition, seven in the imagery condition, two in the implementation intention condition, and one in the combined condition. Body Mass Index (BMI) had five missing data points: one in each of the control, imagery, and implementation intention conditions, and two in the combined condition.

Abbreviation: LIS, leisure index score.

### Intervention manipulations and fidelity checks

Of the 137 participants randomized into one of the intervention conditions, 97 (71%) completed all 4 weeks (63% in the imagery group and 77% in both the implementation intentions and combined conditions).

#### Imagery ability

There were no significant differences in imagery ability between those in the imagery condition or in the combined condition (*F*_(2,120)_ = .815, *p* = .488, ƞ^2^ = .02). Means are presented in Table 1.

#### Implementation intention fidelity

Across both the implementation intention and combined conditions, 96% followed the if‐then instructions; only three participants implementation intentions were not in that format. These three participants were contacted by email (by the first author) with the option to rewrite their implementation intention, with guidance from the researcher, or to withdraw from the study. All three participants adjusted their implementation intentions to ensure they followed the ‘if‐then’ format.

#### Consistency

Results of the mixed RM ANOVA showed no significant interaction (*F*_(6,35)_ = 0.98, *p* = .455, ƞ^2^ = .14) for consistency, but a significant main effect for time (*F*_(2,38)_ = 3.95, *p* = .015, ƞ^2^ = .24) and condition (*F*_(2,39)_ = 3.48, *p* = .041, ƞ^2^ = .15) for consistency. Post hoc testing indicated that for time, consistency was lowest in the first intervention week (0.85 ± .70) compared to all other time points (Ps = .005–.022). For the main effect of condition, post hoc analyses showed those in the imagery only condition (mean .74 ± .12) were significantly less consistent than the implementation intentions only condition (mean 1.08 ± .12, *p* = .022) and combined condition (mean 1.11 ± .14, *p* = .05). Means (SD) are presented in Table 1.

### Effects of condition on habit strength

Results indicated significant main effects of time (*F*_(2,35)_ = 21.94, *p* < .001, ƞ^2^ = .56) and condition (*F*_(3,34)_ = 15.31, *p* < .001, ƞ^2^ = .58) on habit strength. There was also an interaction between time and condition (*F*_(6,31)_ = 6.20, *p* < .001, ƞ^2^ = .55) for habit strength. Follow‐up post hoc tests showed no differences across the three time points for the control condition or the implementation intention condition. The imagery condition had significantly higher habit strength post‐intervention (*t*(69) = −2.82, *p* = .003, *d* = .34) and at follow‐up (mean = 2.42 ± 1.10) compared to baseline (*t*(53) = 3.69, *p* < .001, *d* = .50), but no difference between post‐intervention and follow‐up (*t*(53) = −1.62, *p* = .055, *d* = .22). The combined conditions habit strength was significantly different across all three time points, with post‐intervention habit strength higher than baseline (*t*(52) = −7.78, *p <* 001, *d* = 1.09), and follow‐up habit strength significantly higher than both post‐intervention (*t*(40) = −2.63, *p* = .006, *d* = .41) and baseline (*t*(40) = −8.17, *p* < .001, *d* = 1.34). Means and standard deviations are displayed in Table 1.

Figure 3 displays the changes in weekly habit strength for the intervention conditions. Analyses showed significant main effects of time (*F*_(6,27)_ = 9.74, *p* < .001, ƞ^2^ = .68) and condition (*F*_(2,31)_ = 6.47, *p* = .004, ƞ^2^ = .29) on habit strength, with an accompanying significant interaction between condition and time (*F*_(12,21)_ = 2.72, *p* = .022, ƞ^2^ = .61). Post hoc analysis showed that in the imagery condition, the first significant change in habit strength occurred at post‐intervention (*t*(5) = −3.69, *p* < .001, *d* = .50). For the combined condition a significant increase in habit strength occurred at the end of week three (*t*(52) = −7.71, *p* < .001, *d* = 1.06). There was no difference in habit strength between weeks three and four (*t*(52) = −1.18, *p* = .122, *d* = .516), or post‐intervention (*t*(52) = 1.52, *p* = 0.67, *d* = .21). Habit strength increased significantly at follow‐up (*t*(40) = 3.49, *p* < .001, *d* = .55). No changes in habit strength were found at any time point for the implementation intention group.

### Effects of condition on physical activity

Results of the RM ANOVA for physical activity indicated that there was not a significant interaction between time and condition (*F*_(6,22)_ = 2.34, *p* = .067, ƞ^2^ = .39) and no main effect of condition (*F*_(3,25)_ = 1.92, *p* = .152, ƞ^2^ = .18). There was a significant main effect of time (*F*_(2,26)_ = 18.45, *p* < .001, ƞ^2^ = .58) which indicated a significant decrease in physical activity post‐intervention (*p* < .001, *d* = .98) compared to baseline (Figure 4). Means and standard deviations for physical activity LIS scores are presented in Table 1.

Figure 5 displays the change in physical activity across intervention weeks for the three intervention conditions. There were main effects of time (*F*_(6,14)_ = 8.33, *p* < .001, ƞ^2^ = .78) and condition (*F*_(2,18)_ = 4.67, *p* = .024, ƞ^2^ = .34) for weekly changes in physical activity LIS scores. However, a significant interaction between condition and time was also present (*F*_(11,9)_ = 8.54, *p* = .002, ƞ^2^ = .91). Follow‐up analysis indicated that, in the imagery condition, there is a significant decrease in physical activity at week 1 (*t*(47) = 6.50,*p* = <.001, *d* = .94) from baseline. No other significant weekly changes in physical activity were found for the imagery condition. For the combined condition, there was a significant increase in LIS at week two (*t*(46) = 5.76, *p* < .001, *d* = .75) of the intervention, which continued to increase at weeks three (*t* (46) = 2.90, *p* = .003, *d* = .42) and four (*t* (46) = 3.72, *p* < .001, *d* = .54) of the intervention. A significant decrease in LIS scores at post‐intervention (*t* (46) = −9.14, *p* < .001, *d* = 1.41), compared to week four, was followed by a significant increase in physical activity after the 12‐week follow‐up (*t* (46) = 1.85, *p* = .037, *d* = .31). Complete post hoc results are found in File S2.

## DISCUSSION

**FIGURE 2:**
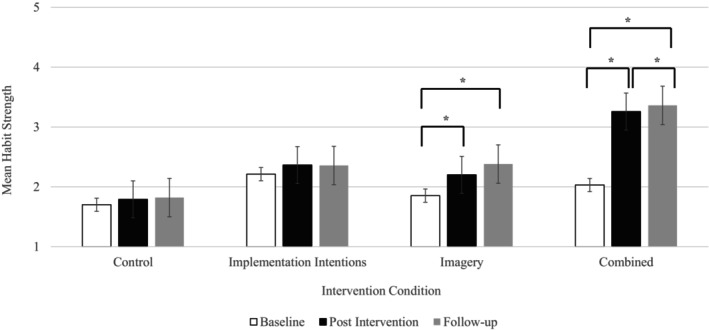
Mean (standard error) habit strength for baseline, post‐intervention and follow‐up time periods by condition.

This preliminary study assessed if implementation intentions would lead to stronger physical activity habits and increased behaviour when reinforced with mental imagery. Overall, results showed that implementation intentions reinforced with mental imagery led to an increase in habit strength after 3 weeks that was maintained at the end of the intervention and at follow‐up 12 weeks later (Figure 2). This is a novel finding, indicating that reinforcing implementation intentions with mental imagery may be associated with greater habit strength faster than the average reported time to form a habit (e.g., Lally et al., 2010).

**FIGURE 3:**
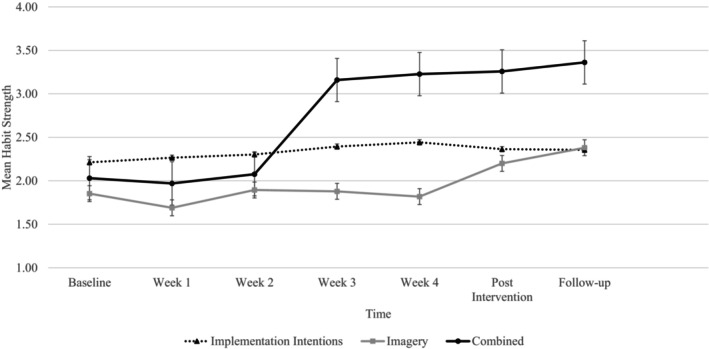
Weekly changes in habit strength for the intervention conditions.

Studies that have tracked habit formation for health behaviours have found it can take between one and 4 months to develop a habit (Kaushal & Rhodes, 2015; Lally et al., 2010) The current findings indicate that the time for habit formation may be shorter, given our data showed that habit strength increased significantly after 3 weeks (see Figure 3) in the group for which implementation intentions were reinforced by imagery. These data suggest that the combined approach may have resulted in automaticity of enacting physical activity and this is likely due to the strengthened mental link between the cue and behavioural response (Adriaanse et al., 2011). The findings also indicate that increases in physical activity started at week two of the intervention and continued to increase throughout the 4 weeks (Figure 5) for the combined group. Taken together, the increases in habit strength and physical activity seen in the combined condition support the assumptions that physical activity repetition increases habit strength, which in turn leads to higher levels of physical activity (Gardner & Lally, 2018). It is suggested that habits develop after a period of repeated behaviour that requires self‐control, which on its own can be difficult to sustain (Hagger et al., 2010). However, the present study suggests that reducing the amount of time to develop automaticity in engaging in physical activity behaviours may reduce drop‐out and support maintenance of physical activity (Figure 2). While the current findings suggest that the combined intervention may support behavioural maintenance, a meta‐analysis has found that effect sizes are larger for interventions that have a follow‐up period of 12 weeks or less compared to studies with a follow‐up period of more than 12 weeks (Ma et al., 2023). Thus, in line with Rebar et al. (2016) and Ma et al. (2023) we recommend that future studies should focus on longer follow‐up periods to establish the impact on the maintenance of habit strength beyond 12 weeks.

While the current findings on the influence of implementation intentions and imagery on habit strength are promising, they need to be discussed with respect to unexpected findings and design limitations. Two key findings in this study are contrary to expectations. Firstly, in the combined condition, physical activity decreased post‐intervention, but habit strength did not. There is some evidence that habit strength does not cease abruptly with some missed opportunities (Lally et al., 2010) and rather decays over time (Edgren et al., 2025) and weak habit strength can persist even four weeks later (Walker et al., 2015). While physical activity decreased during the post‐intervention week, it did not cease completely. Thus, those in the combined condition likely didn't miss every opportunity to be active, thus habit strength wouldn't cease abruptly.

As there was no measurement of habit strength or automaticity across the 12 weeks, there may have been changes in habit strength or automaticity that we were not able to capture. For example, there may have been a drop in habit strength when physical activity was lower, and as people increased their physical activity behaviour, habit strength increased at follow‐up. While the pattern of physical activity behaviour in the combined condition shows that using both imagery and implementation intentions holds promise as an approach to increasing physical activity, further research is needed to determine the impact of the combined approach on increasing physical activity levels.

In addition, participants reported relatively low consistency of their physical activity behaviour. Consistently repeating behaviour in the same context/situation is a critical component of habit formation (Lally & Gardner, 2013). The finding that habit strength increased despite relatively low levels of consistency is contrary to habit theory. We offer several possible explanations for the change in automaticity. First, it could be that the context‐free measure of automaticity used in this study is incongruent with the context‐specific nature of the habit formation intervention provided, such that we simply measured changes in automaticity and not habitual behaviour per se. Context‐free SRBAI summarizes automaticity across all contexts; thus, it is possible that the context‐free measure measured global physical activity habits, which may inflate automaticity scores, rather than the specific physical activity specified in their implementation intention. Indeed, Diefenbacher et al. (2023) found that context‐free automaticity also increased, to a greater extent, than context‐specific automaticity scores following a fruit consumption intervention. Thus, despite low consistency and the reduction in physical activity levels, it is possible that, due to the context‐free measure, automaticity remained for global physical activity across multiple contexts. Second, reporting of low consistency may be due to the focus on repeating behaviour at the same time of day, which may be difficult for people with varying schedules and routines, such as shift work (Kirk & Rhodes, 2011) and those with erratic and changing life circumstances due to illness, new parenthood (Rhodes et al., 2014; Divine et al., 2022), unemployment, or bereavement (Allender et al., 2008). Thus, the opportunity to consistently practice physical activity in the same context may be lower, which may impact the formation of cue‐behaviour connections that are established through consistent repetition (Rhodes & Rebar, 2018).

Third, implementation intentions are thought to influence behaviour through increasing automaticity (Gollwitzer & Sheeran, 2006) meaning that changes in automaticity in the combined condition in this study may indicate that implementation intentions have been effective. However, if this was the case, then we would expect to see changes in automaticity in the implementation intentions only condition, which was not the case in this study (Figures 2 and 3). It is suggested that individual differences in psychological constructs influence the effectiveness of implementation intentions on automaticity and behaviour (Bieleke & Keller, 2021). Specifically, implementation intentions depend on goal commitment or strong intentions (Gollwitzer, 1999; Sheeran et al., 2024), self‐efficacy (Wieber et al., 2010) and are more effective when people are highly motivated (Sheeran et al., 2024) and when motivation is stable (Rhodes et al., 2020). It is possible that people may have been particularly motivated when enrolling in the intervention, and this motivation was not sustained. Lally and Gardner (2013) highlight that habit formation also depends on changes in motivation, capability, or opportunity to act, to support behavioural repetition. Indeed, intrinsic motivation (Hopkins et al., 2022; Gardner & Lally, 2013) and high levels of self‐efficacy (i.e. capability; Stojanovic et al., 2021) are also associated with stronger physical activity habits. Future research is needed to assess the individual differences in goal intention and psychological factors to understand their moderating influence on the effectiveness of habit formation interventions.

Finally, given automaticity increased in both the combined and imagery conditions (Figure 3) but not the implementation intentions only condition, imagery itself may have influenced automaticity directly or indirectly. With respect to the former, in sporting contexts imagery is associated with achieving flow states of performance (Koehn et al., 2014; Nicholls et al., 2005), which are associated with high levels of performance, making the execution of sport‐specific actions easier and automatic (Harris et al., 2017). Imagery may also influence automaticity indirectly by influencing moderators of automaticity, such as self‐efficacy, affect (de Bruijn et al., 2014) and motivation (Hopkins et al., 2022). Research has shown that imagery interventions lead to increases in intention, attitude (Conroy & Hagger, 2018), affective experiences (Weyland et al., 2020), and self‐efficacy beliefs for physical activity (Cumming, 2008; Duncan et al., 2011; Rodgers et al., 2001; Weibull et al., 2015). With respect to the increase in automaticity in the imagery‐only condition, it is surprising that we do not see an increase in physical activity levels in this condition across the intervention and follow‐up. It is not immediately clear why this may be. It is possible that those within this group held low intentions to increase their activity and stayed at similar levels, and the imagery may have simply reinforced the motivational and/or affective components that may be rewarding and influence the automaticity of behaviour at their current level of physical activity.

**FIGURE 4:**
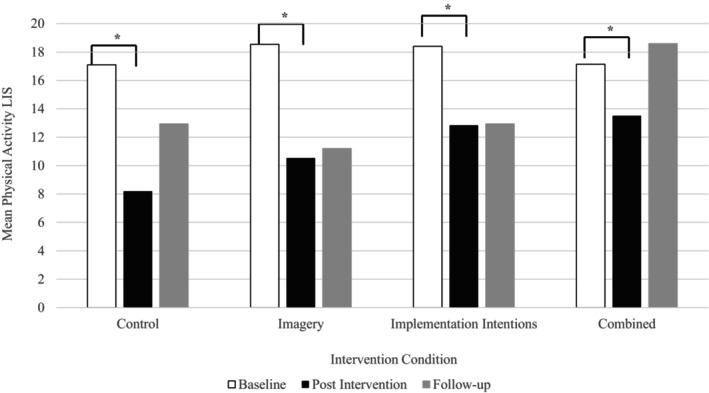
Physical activity levels across time and by condition.

**FIGURE 5:**
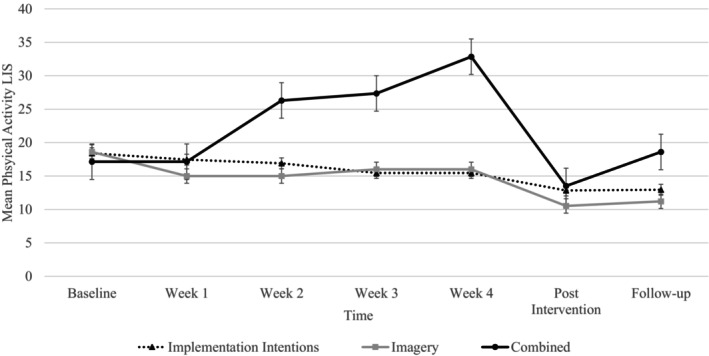
Weekly changes in physical activity for the intervention conditions.

The weekly changes in physical activity in the intervention conditions increased from week two for the combined group (Figure 4). However, results comparing physical activity across control and intervention groups indicated that physical activity levels were not different by group (Figure 5). We saw an unexpected decrease in physical activity post‐assessment in all groups, including the control group, which suggests that the decline may be the result of factors external to the study itself. For instance, data collection post‐assessment occurred just before the UK Easter holidays, which includes at least a four‐day work‐free period for many people, and some take additional holiday during this time. Consistent with this observation, research examining the impact of Easter specifically on the exercise routines of 1210 gym members found that the Easter break can disrupt physical activity, and that this disruption was greater for those with strong habits and more frequency of behaviour (Fredslund & Leppin, 2019). Physical activity levels did increase again at follow‐up to higher levels than those reported at baseline when implementation intentions were reinforced by imagery (Figure 4). This may be the result of increases in automaticity that occurred prior to the decrease in physical activity in this condition. Habit does not cease immediately; rather, it decays over time (Walker et al., 2015) and decreases in habit strength vary substantially between people (Edgren et al., 2025). Thus, after a disruption in encountering the cue or opportunity to perform the behaviour, the previously established habit or automaticity may have made it easier for people to regain their activity levels over time.

A major strength of this study is that it meets the criteria set out by Gardner et al. (2022) for what constitutes a true habit formation study. In addition, strengths of this study include a longitudinal design and novel approaches to physical activity habit formation and methods of intervention delivery. Furthermore, online physical activity habit interventions are more effective for physical activity habit formation than offline delivery (Ma et al., 2023) as they allow for personalization of the interventions to individual preferences. Participants in this study were able to participate in an activity of their own choosing and able to create an implementation intention that fitted their schedule and preferences. As demonstrated by previous research, when participants can freely select their preference, intervention effectiveness on habit formation is greater (Gardner et al., 2023). The use of a 7 days recall self‐report physical activity measure is a potential limitation of the study. While self‐reported physical activity has the capacity to overestimate or underestimate physical activity levels and is subject to reporting biases (social desirability, recall and memory inaccuracies), it is unlikely that any reporting biases would be different across conditions. Future research would benefit from using objective measures of physical activity, such as accelerometers.

Although a longitudinal design with multiple time points allows for strong inferences, moment‐by‐moment changes in habit strength could not be captured. Using ecological momentary assessments may allow for the assessment of habit formation at the individual level to determine individual‐specific trajectories of automaticity change (e.g., N‐of‐1 method: Kwasnicka & Naughton, 2020). Additionally, participants were a self‐selecting sample interested in increasing their physical activity and may have greater motivation than those who do not intend to change; however, this reflects real‐life situations in which people want to change their health behaviours, and those who do not intend to change generally do not (Webb & Sheeran, 2006). However, participants' intentions to be active were not assessed in this study. Furthermore, participants were recruited through a crowdsourcing platform for research and were paid for their participation, which may mean that participants are more digitally literate and that the payment may negatively impact the quality of data. However, research has demonstrated that data collected from research‐centred crowdsourcing platforms are not distinguishable from other ways of recruiting and testing participants and that realistic compensation rates do not affect data quality or reliability (Casler et al., 2013).

This is the first study to assess the impact of implementation intentions and imagery on the formation of physical activity habits. Strategies that accelerate habit formation and promote the maintenance of physical activity are especially important given the benefits of physical activity are not gained immediately but rather accrue with regular long‐term behaviour. This preliminary research suggests promise for a novel approach to the formation of real‐world habits, with our findings indicating that reinforcing specific plans via implementation intentions with mental imagery may facilitate physical activity habit formation. Although further work and replication are needed to assess the robustness of our results, the findings point to the potential of a novel intervention approach that may aid the development of behavioural automaticity and may also decrease the time it takes to strengthen physical activity habits and support the maintenance of behaviour.

## AUTHOR CONTRIBUTIONS

**Alison Divine:** Conceptualization; methodology; investigation; formal analysis; project administration; writing – original draft; writing – review and editing. **Sarah Astill:** Writing – original draft; writing – review and editing.

## Supporting information


Appendix S1.

## Data Availability

The data that support the findings of this study are openly available in University of Leeds Data repository at https://archive.researchdata.leeds.ac.uk/.
