# Supplementary material for: Reinforcing implementation intentions with imagery increases physical activity habit strength and behaviour
Source: Br J Health Psychol. 2025 Mar 18;30(2):e12795. doi: 10.1111/bjhp.12795 (PMC11920387; doi:10.1111/bjhp.12795)
Supplement: Supplementary file 1 — Appendix S1. [file BJHP-30-0-s001.docx]

File S1

Script 1: Imagery condition script

Imagery instructions

Research has shown that you are even more likely to be physically active if you ‘‘mentally image’’ yourself being active in a very vivid manner!

Prior to engaging in imagery, make sure that you are in a comfortable place, that is quite and free from distractions. This session will take you approximately 3 minutes to complete.

We would like you to do a mental imagery exercise in order to help you be physically active. Imagery involves recreating experiences in the mind, including seeing yourself preparing to be active, engaging in the activity and experiencing the movements and emotions as you would if you were physically completing the movement.

Imagine that you are getting ready to be physically active. See yourself getting ready to be active. This may involve changing into different clothing or putting your shoes on. Imagine that you are looking forward to being physically active. See yourself enjoying the activity. Imagine the sensations of physical activity and the environment. For example, if you are outside, notice how the breeze feels and how the grass smells. If you are being active inside, imagine the space you are active in, the sounds, and what you see when being active. Feel your muscles getting warm and feeling strong. See yourself successfully completing the activity. Notice how good you and accomplished you feel afterwards.

Really take a moment to close your eyes and image yourself being physically active in this very detailed and vivid manner! Make sure to use all of your senses as you imagine your physical activity.

**Script 2: Implementation intention condition imagery session**

Please review your implementation intention:

{implementation intention}.

(*Implementation intention inserted automatically through the software)*

Imagery Instructions:

Research has shown that you are even more likely to actually carry out the intention to be physically active if you ‘‘mentally image’’ your implementation intentions in a very vivid manner!

Prior to engaging in imagery, make sure that you are in a comfortable place, that is quite and free from distractions. This session will take you approximately 3 minutes to complete.

We would like you to do a mental imagery exercise in order to help you achieve your goal. Imagery involves recreating experiences in the mind, including seeing yourself preparing to be active, engaging in the activity and experiencing the movements and emotions as you would if you were physically completing the movement.

Image that it is *[time of day/context from implementation intention inserted]* specified in your implementation intention that you want to be active

See yourself getting ready to be active. This may involve changing into different clothing or putting your shoes on. Imagine that you are looking forward to being physically active. See yourself enjoying the activity. Imagine the sensations of physical activity and the environment. For example, if you are outside, notice how the breeze feels and how the grass smells. See yourself successfully completing the activity. Notice how good you and accomplished you feel afterwards.

Really take a moment to close your eyes and image the *[time of day/context from implementation intention inserted]* in this very detailed and vivid manner! Make sure to use all of your senses as you imagine initiating and completing your physical activity

File S2

| Table 1. Means and post hoc paired t-test results for weekly changes in habit strength by condition | | | | | |  |
| --- | --- | --- | --- | --- | --- | --- |
|  | Mean ± SD | Post hoc testing | T-test | P-value | Effect size *d* |  |
| Imagery Condition | | | | | | |
| Baseline | 1.85 ± .92 | Baseline vs Week 1 | t(53) = .55 | .294 | .07 |  |
| Week 1 | 1.84 ± 1.10 | Week 1 vs Week 2 | t(53) = -1.56 | .062 | -.20 |  |
| Week 2 | 1.94 ± 1.25 | Week 2 vs Week 3 | t(53) = -.27 | .396 | -.04 |  |
| Week 3 | 1.86 ± 1.22 | Week 3 vs Week 4 | t(53) = .45 | .326 | .06 |  |
| Week 4 | 1.83 ± 1.37 | Week 4 vs Post | t(53) = -2.51 | .007 | -.31 |  |
| Post | 2.20 ± 1.27 | Post vs Follow-up | t(53) = -1.62 | .055 | -.22 |  |
| Follow up | 2.38 ± 1.11 |  |  |  |  |  |
| Implementation intentions condition | | | | | | |
| Baseline | 2.04 ± .1.01 | Baseline vs Week 1 | t(58) = -1.40 | .084 | -.18 |  |
| Week 1 | 2.18 ± .94 | Week 1 vs Week 2 | t(58) = .48 | .317 | .06 |  |
| Week 2 | 2.14 ± .98 | Week 2 vs Week 3 | t(58) = -.56 | .328 | -.07 |  |
| Week 3 | 2.18 ± 1.09 | Week 3 vs Week 4* | t(58) = -1.44 | .078 | -.19 |  |
| Week 4 | 2.21 ± 1.09 | Week 4 vs Post | t(58) = .74 | .231 | .10 |  |
| Post | 2.19 ± 1.10 | Post vs Follow-up | t(43) = -.13 | .448 | -.02 |  |
| Follow up | 2.14 ± 1.03 |  |  |  |  |  |
| Combined condition | | | | | | |
| Baseline | 2.03 ± 1.04 | Baseline vs Week 1 | t(52)= -1.18 | .122 | -.16 |  |
| Week 1 | 2.07 ± 1.00 | Week 1 vs Week 2 | t(52)= -1.15 | .128 | -.16 |  |
| Week 2 | 2.21 ± 1.03 | Week 2 vs Week 3 | t(52) = -6.11 | <.001 | -.84 |  |
| Week 3 | 3.13 ± .70 | Week 3 vs Week 4 | t(52) = -1.18 | .122 | -.16 |  |
| Week 4 | 3.18 ± .10 | Week 4 vs Post | t(52) = -1.00 | .161 | -.14 |  |
| Post | 3.22 ± .67 | Post vs Follow-up | t(40) = -2.63 | .006 | -.41 |  |
| Follow up | 3.36 ± .63 |  |  |  |  |  |
| Note: * t-test cannot be computed because the standard error of the difference is 0 as the means and standard deviations are the same at week three and four time points. | | | | | | |

| Table 2. Means and post hoc paired t-test results for weekly changes in physical activity by condition | | | | | |  |
| --- | --- | --- | --- | --- | --- | --- |
|  | Mean ± SD | Post hoc testing | T-test | P-value | Effect size *d* |  |
| Imagery Condition | | | | | | |
| Baseline | 18.55 ± 7.78 | Baseline vs Week 1 | t(47) = 6.50 | <.001 | .94 |  |
| Week 1 | 8.63 ± 13.57 | Week 1 vs Week 2 | t(47) = -1.72 | .046 | -.25 |  |
| Week 2 | 10.94 ± 15.83 | Week 2 vs Week 3 | t(46) = -.06 | .477 | -.01 |  |
| Week 3 | 11.02 ± 13.75 | Week 3 vs Week 4* |  |  |  |  |
| Week 4 | 11.02 ± 13.75 | Week 4 vs Post | t(47) = .31 | .379 | .05 |  |
| Post | 10.53 ± 15.44 | Post vs Follow-up | t(52) = -.27 | .394 | -.04 |  |
| Follow up | 11.21 ± 18.09 |  |  |  |  |  |
| Implementation intentions condition | | | | | | |
| Baseline | 18.41 ± 6.88 | Baseline vs Week 1 | t(56)=1.49 | .071 | .20 |  |
| Week 1 | 14.46 ± 15.55 | Week 1 vs Week 2 | t(49)= -1.21 | .116 | -.17 |  |
| Week 2 | 16.4 ± 17.31 | Week 2 vs Week 3 | t(47) = -.01 | .496 | -.00 |  |
| Week 3 | 16.31 ± 18.84 | Week 3 vs Week 4* |  |  |  |  |
| Week 4 | 16.31 ± 18.84 | Week 4 vs Post | t(47) = 1.34 | .093 | .20 |  |
| Post | 12.84 ± 13.09 | Post vs Follow-up | t(43) = -.06 | .476 | -.01 |  |
| Follow up | 12.95 ± 13.09 |  |  |  |  |  |
| Combined condition | | | | | | |
| Baseline | 17.14 ± 4.67 | Baseline vs Week 1 | t(48)=.16 | .436 | .02 |  |
| Week 1 | 15.94 ± 12.6 | Week 1 vs Week 2 | t(47)= -5.19 | <.001 | -.75 |  |
| Week 2 | 25.13 ± 15.07 | Week 2 vs Week 3 | t(46) = -2.90 | .003 | -.43 |  |
| Week 3 | 27.66 ± 15.76 | Week 3 vs Week 4 | t(46) = -3.72 | <.001 | -.42 |  |
| Week 4 | 35.4 ± 16.45 | Week 4 vs Post | t(41) = 9.14 | <.001 | 1.41 |  |
| Post | 13.51 ± 16.8 | Post vs Follow-up | t(34) = -1.85 | .037 | -.31 |  |
| Follow up | 18.6 ± 16.14 |  |  |  |  |  |
| Note: * t-test cannot be computed because the standard error of the difference is 0 as the means and standard deviations are the same at week three and four time points. | | | | | | |
